# Mycoplasma pneumoniae and Adenovirus Coinfection Cause Pediatric Severe Community-Acquired Pneumonia

**DOI:** 10.1128/spectrum.00026-22

**Published:** 2022-03-21

**Authors:** Fei Li, Yuhan Zhang, Peng Shi, Linfeng Cao, Liyun Su, Pan Fu, Kuerbanjiang Abuduxikuer, Libo Wang, Yin Wang, Roujian Lu, Wenjie Tan, Jun Shen

**Affiliations:** a Department of Infectious Diseases, Children’s Hospital of Fudan University, National Children’s Medical Center, Shanghai, China; b Statistics and Data Management Center, Children’s Hospital of Fudan University, National Children’s Medical Center, Shanghai, China; c Department of Virology, Children’s Hospital of Fudan University, National Children’s Medical Center, Shanghai, China; d Department of Clinical Microbiology Laboratory, Children’s Hospital of Fudan University, National Children’s Medical Center, Shanghai, China; e Department of Respiratory, Children’s Hospital of Fudan University, National Children’s Medical Center, Shanghai, China; f Clinical Trial Unit, Children’s Hospital of Fudan University, National Children’s Medical Center, Shanghai, China; g National Institute for Viral Disease Control and Prevention, China CDC, Beijing, China; Wright State University

**Keywords:** severe community-acquired pneumonia, consolidation, pathogen, alveolar lavage fluid, multiple polymerase chain reaction, children

## Abstract

Consolidation is one complication of pediatric severe community-acquired pneumonia (SCAP) that can respond poorly to conservative medical treatment. We investigated the pathogens that cause pediatric SCAP including cases with persistent consolidation that need bronchoscopy intervention. Alveolar lavage fluid (ALF) samples collected from cases admitted to Children’s Hospital of Fudan University with SCAP during January 2019 to March in 2019 were retrospectively tested by the RespiFinder 2SMART multiplex PCR (multi-PCR) assay targeting 22 respiratory pathogens. A total of 90 cases and 91 samples were enrolled; 80.0% (72/90) of the cases had pulmonary consolidation and/or atelectasis. All samples were positive with targeted pathogens tested by multi-PCR, and 92.3% (84/91) of the samples were co-detected with pathogens. Mycoplasma pneumoniae (MP) and *adenovirus* (ADV) as the two dominant pathogens, with the positive rates of 96.7% (88/91) and 79.1% (72/91), respectively. Most of the samples were positive with MP and ADV simultaneously. As a control, 78.0% (71/91) of the samples were positive by conventional tests (CT), in which MP had the detection rate of 63.9% (55/86) by a traditional real-time PCR assay, while ADV were positive in 13.1% (12/91) of the samples by a direct immunofluorescence assay (DFA). In cases with persistent pulmonary consolidation, the positive rates of pathogens by multi-PCR and CT were 100% (72/72) and 81.9% (59/72), respectively. There were no significant differences of MP or ADV positive rates between cases with and without pulmonary consolidation. MP and ADV most prevalent in pediatric SCAP cases required fiberscope intervention, and presented with coinfections dominantly.

**IMPORTANCE** Pathogens that cause pediatric severe community-acquired pneumonia (SCAP) requiring bronchoscopy intervention are understudied. Through this study, we explore the etiology of SCAP form alveolar lavage fluid (ALF) samples by the RespiFinder 2SMART multi-PCR assay. It is observed that high mixed detection rates of Mycoplasma pneumoniae and *adenovirus* in ALF samples collected from hospitalized SCAP children experienced bronchoscopy intervention. Eighty percent of the cases had pulmonary consolidation and/or atelectasis. The presence of possible coinfection of these two pathogens might contribute to poor clinical anti-infection response. The results of this study might be helpful for the selection of clinical strategies for the empirical treatment of such pediatric SCAP cases.

## INTRODUCTION

Respiratory viruses, bacteria including Mycoplasma pneumoniae (MP), are dominant pathogens that cause pediatric community-acquired pneumonia (CAP) (1–7). Previous studies had proven that MP and other pathogens cause coinfection in children with pneumonia (6–9). The existence of pulmonary coinfection is always accompanied by the increase of the severity of the disease and the complexity of treatment (8–10). Consolidation and lung abscesses caused by mixed pulmonary pathogens can present as poor efficacy of antimicrobial therapy in the acute stage, and relate with complications including pleural effusion, necrotizing pneumonia, and even higher mortality (11–15). Our previous studies reported that MP and *adenovirus* (ADV) were important pathogens of pediatric pneumonia in Shanghai (16–18). However, the identification of pathogens in severe community-acquired pneumonia (SCAP) pediatric patients was unclear, especially in cases that required bronchoscopy intervention. Here we reported the results of detecting 22 common pneumonia pathogens from alveolar lavage fluid (ALF) samples by the RespiFinder 2SMART multi-PCR assay in pediatric patients with SCAP needing bronchoscopy intervention in Shanghai.

## RESULTS

### Clinical characteristics of patients.

A total of 99 children who experienced fiberscope intervention during the study period, and 100 ALF samples were obtained. Nine cases were excluded including three cases with inhaled foreign bodies, two cases with nosocomial pneumonia, and four without enough samples for multi-PCR. Finally, 91 specimens from 90 cases met the inclusion criteria. Of these, 51 were males and 39 were females. The mean age was 4.9 ± 3.2 years, with a range from 0.4 to 13.0 years. Eighty percent (80%, 72/90) of the patients had pulmonary consolidation and/or atelectasis, and 28.8% (26/90) had pleural effusion (Data set S2, Data set S3). All 72 cases presented with persistent lung consolidation and a history of more than 3 days of azithromycin or erythromycin anti-infection. Meantime, 13.3% (12/90) of patients had underlying diseases: four had immune deficiency (two had agammaglobulinemia, one had chronic granulomatous disease, and one had PIK3CD gene mutation), while the other eight patients each had various underlying conditions such as chronic diarrhea with pancreas exocrine insufficiency, epilepsy, tuberous sclerosis, Prader-Willi syndrome, congenital bronchial stenosis muscular torticollis, bronchial asthma, congenital malformation (single kidney deficiency), and congenital heart disease (postoperative). None of the 90 cases required invasive mechanical ventilation, and all the cases responded poorly to macrolides treatment more than 3 days.

### RespiFinder 2SMART multi-PCR results.

**FIG 1:**
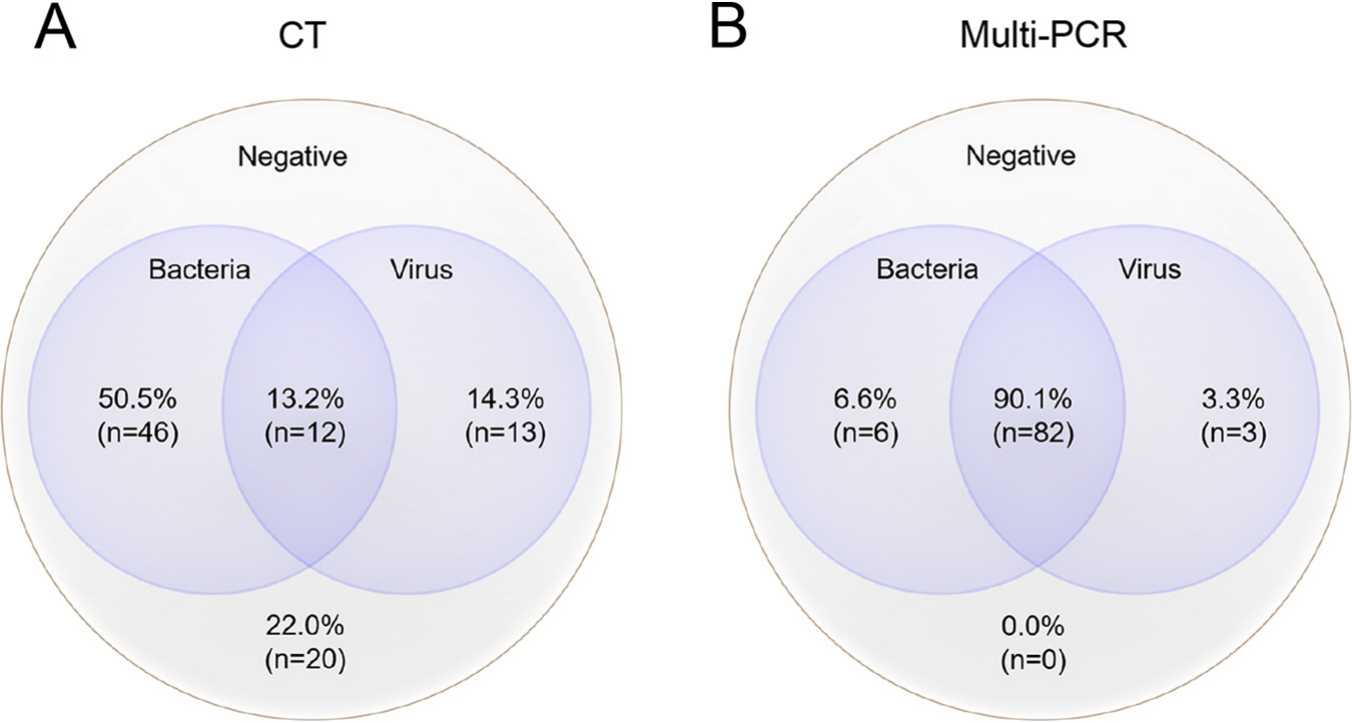
Mixed pathogens detection by CT and multi-PCR. (A) The positive rate of pathogens by CT was 78.0% (71/91). Bacteria were found in 63.7% (58/91) of the samples, including MP detected from 63.9% (55/86) of the samples. The targeted viruses were positive in 27.5% (25/91) of the samples. The co-positive rate of pathogens of bacteria and viruses was 13.2% (12/91). Twenty samples were negative tested by CT. (B) All (91/91, 100.0%) samples were positive tested by multi-PCR. The positive rate of MP and targeted viruses were 96.7% (88/91) and 87.9% (85/91), respectively. The co-positive rate of bacteria and viruses was 90.1% (82/91).

**FIG 2:**
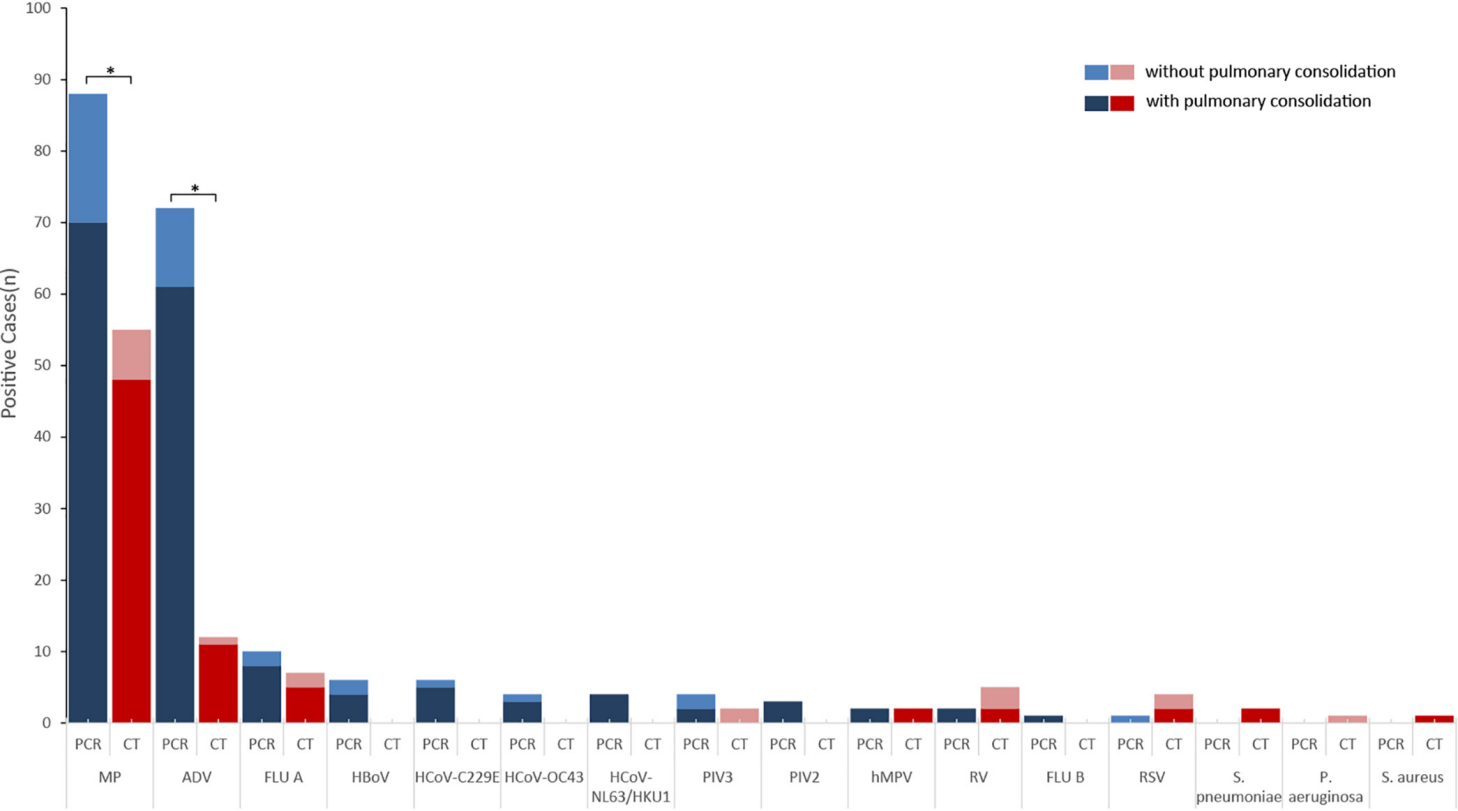
Pathogen detection of the 90 cases by RespiFinder 2SMART multi-PCR. The results of 2009 influenza A (H1N1) pandemic positive in RespiFinder 2SMART multi-PCR were counted as FLUA. One 2-year-old patient had two specimens, his first ALF sample was positive with MP and ADV, he had the second fiberscope intervention 3 days later and the ALF sample was reported as MP, ADV, and HCoV-C229E positive. “*”, pathogens positive rate of 91 samples by multi-PCR compared with that of CT, *P < *0.05.

**FIG 3:**
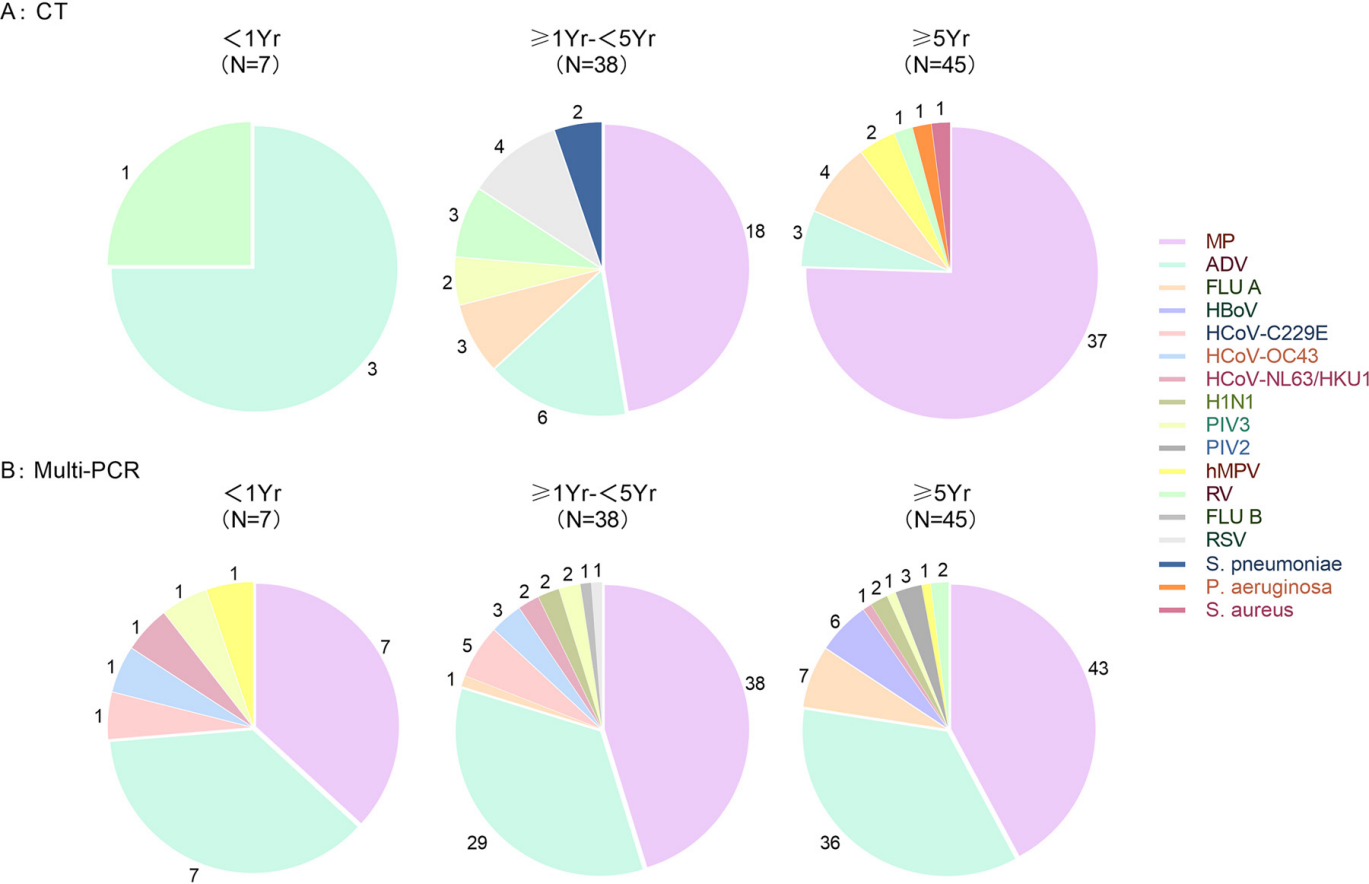
Pathogens detected in 90 cases by CT and multi-PCR in three groups.

Screened by the multi-PCR, 100% (91/91) of ALF samples were positive with at least one pathogen (Fig. 1, Table 1). MP and ADV were the two most frequently detected pathogens, with positive rates of 96.7% (88/91) and 79.1% (72/91), respectively. Mixed pathogens were detected in 92.3% (84/91) of the samples (Table 2). MP was found in 95.8% (69/72) of the samples from the cases with pulmonary consolidation and/or atelectasis. None of the samples were positive with Chlamydia pneumoniae, Legionella pneumophila, and Bordetella pertussis. In cases without pulmonary consolidation, MP was found in 94.7% (18/19) of the samples; there was no significant difference of MP positive rates between cases with and without pulmonary consolidation (*P = *1.00). Meanwhile, the positive rates of ADV in the samples from children with and without pulmonary consolidation or atelectasis were 84.7% (61/72) and 57.8% (11/19), respectively (*P = *0.056) (Fig. 2). Both MP and ADV were the two dominate pathogens among the three ages groups (Fig. 3).

**TABLE 1:** The 8 viruses in 91 samples detected by DFA in CT and multi-PCR

Target pathogen	No. (%) of positive by multi-PCR	No (%). of positive by DFA	No of both positive by multi-PCR and DFA	*χ^2^*	*P* value
Total	80/91(87.9)	21/91(23.0)		77.441	<0.001
ADV	72(79.1)	12(13.1)	11	79.592	<0.001
FLU A	8(8.7)	7(7.6)	1	0.073	0.788
FLU B	1(1.0)	0	0		>0.999^*a*^
RSV	1(1.0)	4(4.3)	1		0.368^*a*^
hMPV	2(2.1)	2(2.1)	0		>0.999
PIV1	0（0.0）	0（0.0）	0（0.0）		
PIV2	3(3.2)	0（0.0）	0（0.0）		0.246^*a*^
PIV3	4(4.3)	2(2.1)	1		0.682^*a*^

a *P*-value was estimated by Fisher’s exact test. Double positive of the eight viruses were found in 14 samples by multi-PCR, and in 6 samples by CT.

**TABLE 2:** Pathogens in 91 samples detected by multi-PCR and CT^*a*^

Pathogen	No. of multi-PCR/CT	Pathogens	No. of multi-PCR/CT	Pathogens	No. of multi-PCR/CT	Pathogens	No. of multi-PCR/CT
MP	6/42	MP, ADV	47/2	MP, ADV, FLUA	1/2	MP, ADV, PIV3, HCoV-C229E	1/0
ADV	1/5	MP, RV	1/2	MP, RV, PIV3	1/0	MP, ADV, FLUA, H1N1-2009	1/0
RV/EV	0/2	MP, FLUA	2/2	ADV, FLUA, hMPV	0/0	MP, FLUA, HBoV, HCoV-NL63/HKU1	1/0
RSV	0/1	MP, hMPV	1/2	MP, FLUA, RSV	0/1	MP, FLUA, HBoV, H1N1-2009	1/0
FLUA	0/2	MP, PA	0/1	MP, ADV, RSV	0/1		
SP	0/2	ADV, RSV	1/1	MP+ADV+PIV2	3/0		
SA	0/1	ADV, PIV3	0/1	MP, ADV, HCoV-C229E	3/0		
		MP, HCoV-OC43	2/0	MP, ADV, HCoV-OC43	2/0		
		MP, HCoV-C229E	2/0	MP, ADV, HCoV-NL63/HKU1	2/0		
		ADV, FLUA	1/0	MP, ADV, H1N1-2009	2/0		
		ADV, HCoV-NL63/HKU1	1/0	MP, ADV, HBoV	3/0		
		RV, PIV3	0/1	MP, ADV, PIV3	2/0		
				MP, ADV, FLUB	1/0		
				MP, ADV, hMPV	1/0		
				MP, FLUA, HBoV	1/0		
Unique pathogen (*n* = 7/55)		Dual pathogens (*n* = 58/12)		Triple pathogens (*n* = 22/4)		Quadruple pathogens(*n* = 4/0)	

a PA, Pseudomonas aeruginosa; SA, Staphylococcus aureus; SP, Streptococcus pneumonia. The multi-PCR targeted pan-RV/EV.

### CT results.

Seventy-one (71/91, 78.0%) samples were positive with at least one pathogen from CT reports (Fig. 1, Table 3). The eight common viruses (respiratory syncytial virus [RSV], adenovirus, Influenza [ADV], Influenza B [FLUB], Parainfluenza 1-3 [PIVI-3], human metapneumovirus [hMP]) were found in 23.0% (21/91) of the samples by direct immunofluorescence assay (DFA), including six samples that were double positive. The positive rates of ADV, FLUA, RSV, FLUB, PIV1-3, and hMPV were 13.1% (12/91), 7.6% (7/91), 4.3% (4/91), 0, 2.1% (2/91), and 2.1% (2/91), respectively (Table 1). From the real-time PCR test assay, 63.9% (55/86) of the samples were MP positive. Rhinovirus (RV) was found in five samples, including one sample that had RV and PIV3, two samples had RV and MP. Sixteen (16/91,17.5%) samples were reported as mixed pathogens being detected, including 4.3% (4/91) of samples that had more than two pathogens (Fig. 1, Table 2). Additionally, each strain of Streptococcus pneumoniae, Pseudomonas aeruginosa, and Staphylococcus aureus was isolated from three samples (Fig. 2). In seven samples from seven cases younger than 1 year, three were positive with ADV, and three were positive with RV. In cases 1 year old and older, MP and ADV were the two dominate pathogens detected, which were consistent with the results of multi-PCR (Fig. 3).

### Comparison of multi-PCR and CT.

There was a significant difference between the multi-PCR and CT in the total pathogens positive rates, with 100% (91/91) and 78.0% (71/91), respectively (*P < *0.001). Mixed pathogens were more frequently detected by the multi-PCR assay (84/91, 92.3%) than CT (16/91, 17.6%) (*P < *0.001). Multi-PCR assay was more sensitive to detect the eight viruses than DFA; the positive rates were 87.9% (80/91) and 23.0% (21/91), respectively (*P < *0.001). The multi-PCR was more sensitive to detect ADV in the specimens (*P < *0.001), while there were no differences in the detection of the other seven viruses (RSV, FLUA, FLUB, PIV1-3, and hMPV) between the two methods (Fig. 3). Five ALF samples had not been sent to real-time PCR test for some reasons, and the detection rates of MP by multi-PCR and the real-time PCR were 96.7% (88/91) and 63.9% (55/86), respectively (*P < *0.001). With respect to pulmonary consolidation, the detection rates of pathogens by multi-PCR and CT were 100% (72/72) and 81.9% (59/72), respectively (*P < *0.001). The sensitivity and specificity of the multi-PCR compared with CT are presented in Table S1 to S3 (Data set S4).

## DISCUSSION

Mycoplasma pneumoniae is an atypical bacteria that can cause life-threatening respiratory tract infections in children (2, 8, 9). During the past several years, there were more world-wide reports concerning infections caused by this organism, including macrolide resistance strains that manifest as refractory mycoplasma pneumoniae infection (19). The present study has two significant findings. First, high incidence of MP with ADV coinfections were found in children with SCAP who required bronchoscopy intervention. Second, there was no significant difference of MP and ADV positive rates between cases with and without pulmonary consolidation in those patients.

We previously demonstrated that different respiratory pathogens can be detected simultaneously in respiratory specimens of pneumonia in children (16–18). In this study, we used a multi-PCR compared with CT to detect pathogens from 91 ALF samples collected by bronchoscopy. The pathogen positive rate was up to 78.0% (71/91) in CT, close to the result of 81.0% (1802/2222) from a large multi-center prospective study conducted in the United States (1). We found MP was the most detected pathogen (55/86, 63.9%), followed by ADV (12/91, 13.1%) and FLUA (7/91, 7.6%) in CT. Similar findings have been reported by other Chinese scholars detected by a high-throughput GeXP-based multiplex PCR assay (20). Meanwhile, the detection rates of MP, ADV, and FLUA by multi-PCR were as high as 96.7% (88/91), 79.1% (72/91), and 8.7% (8/91), respectively. Such high detection rates of MP and AVD from ALF samples has also been reported in previous studies based on next-generation high-throughput sequencing technologies (17, 21). Hence, both the results of CT and multi-PCR had proved MP, ADV, and FLUA were the most common pathogens detected from the patients who received fiberscope intervention, consistent with previous findings (17, 20–23). Additionally, we found that the total positive rate of MP-IgM in these cases were 63.7% (51/80), and supported the PCR results to a certain extent (Data set S2). Considering that MP-IgM would consistently be positive for several months after the onset of acute infection, we did not include it in the CT indicators. But, the detection rates of MP and ADV in our study that were much higher than most previous studies might be related to the following reasons: the selected cases were relatively severe, there was an outbreak of MP and ADV during this study period, and the nucleic acid of MP could exist for a long time in the airway (23).

Briefly, this study provided information that the possibility of coinfection of MP and ADV should be considered in the initial empirical treatment strategy of pediatric SCAP.

In terms of the distribution of pathogens, the eight common respiratory viruses (ADV, RSV, FLUA, FLUB, PIV1-3, and hMPV) were the main infectious agents of these cases. Possibly because the multi-PCR was more sensitive to ADV, the detection rate of the eight common respiratory viruses in 91 specimens was significantly higher than that of CT, although there were no differences in the other seven viruses. Beckmann et al. reported that, when comparing with Luminex NxTAG-Respiratory Pathogen Panel, the sensitivity and specificity of RespiFinder-22 for ADV were 100% and 99.6%, respectively (24).

Another interesting finding of our study was the extremely high nucleic acid detection rate of MP. MP infection can cause airway obstruction, producing exotoxin named community-acquired respiratory distress syndrome toxin (CARDS TX) resulting in cell swelling, nuclear lysis, mucus proliferation, and cell vacuolization (25). We noted 80.0% (72/90) of the cases in our study had pulmonary consolidation, and the positive rate of MP between the cases with (69/72, 97.2%) and without (18/19, 94.7%) pulmonary consolidation had no significant statistically difference (*P* = 1.00). That result exceeded our original expectations. Meanwhile, the positive rates of ADV in the samples from children with and without pulmonary consolidation or atelectasis were 84.7% (61/72) and 57.8% (11/19), respectively (*P = *0.056). We reviewed the reasons for bronchoscopy intervention in those cases, and all cases had objective reasons for poor efficacy of anti-infection treatment before bronchoscopy intervention. Concerns about airway malformation have also been documented in the history of some cases.

The RespiFinder 2SMART multi-PCR assay was designed to target pathogens such as Chlamydia pneumoniae, Legionella pneumophila, and Bordetella pertussis, but there are no positive finding in our study, possibly because of the limited samples size of this study. We should mention that the multi-PCR assay could not detect Streptococcus pneumoniae, Haemophilus influenzae, Pseudomonas aeruginosa, and other common respiratory bacteria, which are important pathogens of pneumonia in children (1, 2, 26). There were two strains of Streptococcus pneumoniae, and each one strain of Pseudomonas aeruginosa and Staphylococcus aureus were isolated by traditional bacterial culture in the 91 samples. The low detection rate of bacterial pathogens might be related to the use of antibiotics before specimen collection. Another possibility was that bacterial infections might be infrequent in those cases. Liu et al. reported that coinfection of bacterial in patients with refractory mycoplasma pneumoniae pneumonia was rare, detected by traditional bacterial culture and next-generation sequencing (27).

In 2012, the U.S. Food and Drug Administration (FDA) approved multi-PCR for clinical detection of respiratory infection, which has been proved more sensitive than CT to test nasopharyngeal samples, and the FilmArray Pneumonia Panels were recommended for the diagnosis of pneumonia pathogens (28). Furthermore, the excellent performance of RespiFinder 2SMART Multiplex PCR (multi-PCR) has been recorded in clinical studies (18, 24, 29, 30).

Our study has some limitations. First, this is a retrospective study from a single center; we are not sure whether similar pediatric cases in Shanghai region at the same time had such detection rates of MP and ADV. Second, we did not sequence the MP and ADV strains to compare the clinical manifestations of pulmonary consolidation with genotype differences. Most importantly, in the absence of “pathogens culture” as the golden standard described in Koch’s postulates, whether it could be used as “the second golden standard” based on highly sensitive nucleic acid tests was controversial.

In conclusion, both CT and the RespiFinder 2SMART multi-PCR assay proved that MP and ADV were the two dominate pathogens detected from pediatric SCAP ALF samples in this study. The presence of the mixed infection of these two pathogens might lead to poor clinical anti-infection treatment and therefore bronchoscopy intervention. At the same time, there were no differences in the detection rates of these two pathogens in those patients with or without pulmonary consolidation.

## MATERIALS AND METHODS

### Enrollment.

ALF samples obtained from patients who experienced fiberscope intervention from January 1, 2019 to March 31, 2019 in Children’s Hospital of Fudan University were retrospectively tested. The medical records of those patients were reviewed. Patients were enrolled according to the inclusion criteria: (i) <18 years old; (ii) diagnosis with SCAP according to the diagnostic criteria recommended by the 2011 Infectious Diseases Society of America community-acquired pneumonia management guideline, including cases needing invasive mechanical ventilation, with multi-lobar infiltrates, or presence of effusion, etc. (26); and (iii) fiberscope intervention was performed and adequate samples of ALF (≥0.5 mL) were preserved. Patients without infectious pneumonia, nosocomial acquired pneumonia, neonates, aspiration pneumonia, including airway foreign body inhalation, and tuberculosis infections were excluded. Samples without enough volume (<0.5 mL) to complete multi-PCR were also excluded.

### Ethical approval.

Our study involving human participants was reviewed and approved by the medical ethics committee of the Children’s Hospital of Fudan University (2020-209).

### RespiFinder 2SMART multi-PCR testing.

The nucleic acid was extracted using a QIAamp MinElute Virus Spin Kit (Qiagen, Hilden, Germany). The two rounds of multi-PCR was performed by LightCycler 480 PCR instrument (Roche, Basel, Switzerland). Reverse transcription (RT) was carried out as follows: 50°C for 10 min; 95°C for 2 min. Multiplex PCR was performed in following steps: step 1, 40 cycles of 94°C for 20 s, 55°C for 20 s, and 72°C for 35 s; step 2, 95°C for 2 min; 10 cycles of 94°C for 20 s, 55°C for 15 s, and 72°C for 15 s; step 3, 23 cycles of 94°C for 15 s, 50°C for 15 s, and 72°C for 15 s; 95°C for 2 min; step 4, 40°C for 90 s; 95°C for 2 min; 37°C for 1 s. Quality control was set to follow the manual by confirming the amplification of the target and the control based on the melting curves (Data set S1). The multi-PCR kit for 22 respiratory pathogens and operation manual were supplied by GeneoDx Biotech Co, Shanghai, China, which cooperated with the Netherlands’ company (Table 3). Compared with the Luminex NxTAG-Respiratory Pathogen Panel, the sensitivity and specificity of the RespiFinder 2SMART multi-PCR were more than 97.7% and 87.5%, respectively(24). And the RespiFinder 2SMART multi-PCR had higher sensitivity and capability to detect 22 pathogens compared to 14 with the RealAccurate Respiratory RT PCR Kit (29).

**TABLE 3:** RespiFinder 2SMART multi-PCR for 22 respiratory pathogens

Virus	Bacteria
Influenza A/B	Mycoplasma pneumoniae
Respiratory syncytial virus A/B	Chlamydia pneumoniae
Human metapneumovirus	Legionella pneumophila
Rhinovirus/enterovirus	Bordetella pertussis
Adenovirus	
2009 influenza A (H1N1) pandemic	
Parainfluenza 1/2/3/4	
Coronavirus NL63/HKU1/229 E/OC43	
Bocavirus	

### Review of clinical microbiology laboratory detection results.

All ALF samples were sent to the clinical microbiology laboratory after being collected. Respiratory syncytial virus (RSV), ADV, influenza A (FLUA), influenza B (FLUB), and Parainfluenza virus 1–3 (PIV1-3) were detected by a DFA assay (Chemicon Respiratory Virus Diagnostic Kit, Chemicon International Inc, Temecula, CA, USA). Human metapneumovirus (hMPV) were detected by a separated DFA assay (Diagnosis Hybrids Diagnostic Kit, Diagnosis Hybrids Inc, USA). RV was detected by a RT-PCR assay (PCR-fluorescent probe for RV, Hubei Langde Medical Technology Co. LTD, China). MP-DNA was detected by a fluorescent real-time PCR assay (DaAn Gene Co. Ltd, Guangzhou, China). All ALF samples were routinely sent for traditional bacterial culture after being collected. The results of the hospital clinical microbiology laboratory were reviewed by our medical history database.

### Statistical analysis.

Continuous variables were presented as mean ± standard deviation (SD), and compared by Student’s *t* test or median (inter-quartile range [IQR]), and non-parametric tests according to the distribution of the data. Categorical variables were expressed as number (%) and analyzed by χ^2^ tests or Fisher’s exact tests when appropriate. The SPSS 23.0 was used to analyze the data and the significance level was set at *P < *0.05.

### Data availability.

The data sets used and/or analyzed during the current study are available from the corresponding author on reasonable request. The names of the repository/repositories and accession number(s) can be found in the supplementary material.
