# Supplementary material for: Mycoplasma pneumoniae and Adenovirus Coinfection Cause Pediatric Severe Community-Acquired Pneumonia
Source: Microbiol Spectr. 2022 Mar 21;10(2):e00026-22. doi: 10.1128/spectrum.00026-22 (PMC9045297; doi:10.1128/spectrum.00026-22)
Supplement: SUPPLEMENTAL FILE 1 — Supplemental material. Download SPECTRUM00026-22_Supp_1_seq7.pdf, PDF file, 0.4 MB [file spectrum00026-22_supp_1_seq7.pdf]

Figure Legend

Supplemental Figure. Quality control of RespiFinder 2SMART Multi-PCR. Quality control was set fellow the manual by confirmation the amplification of the target and the control based on the melting curves.

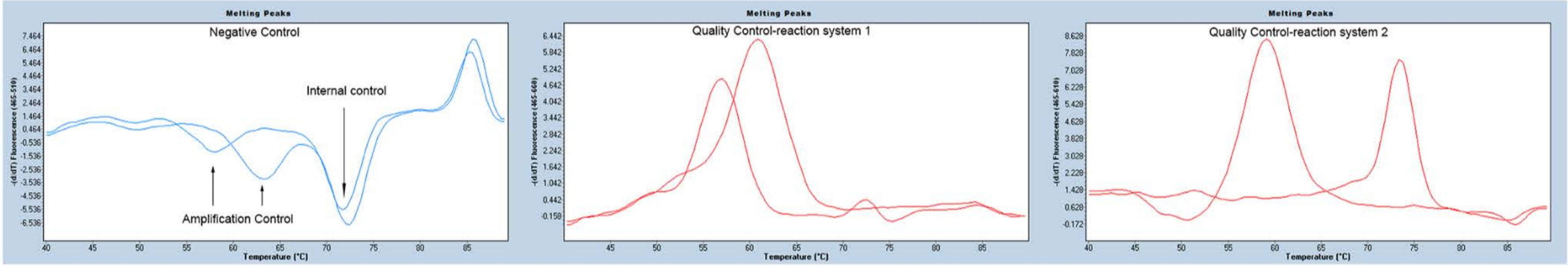

Negative control and quality control of the multiplex PCR system

Clinical information of 90 cases													
Sample ID	Month	Gender:1 for male, 2 for female	Age(year)	Discharged with Diagnosis	Imaging:1 for the chest X-ray, 2 for CT scan-chest：	Multiple PCR results	DFA（1.RSV 2.FLU A 3.FLU B 4.PIV1 5.PIV2 6.PIV3 7.ADV 8.hMPV)	Real-time RT-PCR for rhinovirus (RV)	Real-time PCR for Mycoplasma pneumonia（MP)	Serum antibodies against M. hyopneumoniae(MP-IgM)	AlveolarLavage Fluid Culture	Underlying diseases
S1	1	2	8.6	Severe pneumonia, lung consolidation, pleural effusion, refractory mycoplasma infection, liver damage, hypoproteinemia	2.Left lower lung pneumoniai, right lung consolidation, pleural effusion	hMPV、MP	0	0	> 1.00E+08	1:1280	0	0
S2	1	2	1.2	Interstitial pneumonia, EBV/CMV infection, rhinovirus infection, mycoplasma pneumoniae infection, thrombocytopenia, immunodeficiency?	1Interstitial pneumonia .2.Interstitial pneumonia	MP、HCoV-OC43	0	1	4.48E+03	1:160	0	0
S3	1	2	9.3	Pneumonia	1.Left lung pneumonia. 2.Left lung pneumonia	ADV、MP	0	0	1.13E+06	1:1280	0	0
S4	1	2	7	Mycoplasma pneumoniae pneumonia, lung consolidation	1.Right lung pneumoniae	ADV、MP	0	0	2.48E+07	1:1280	0	0
S5	1	2	6.7	Mycoplasma pneumoniae pneumonia, lung consolidation	1.Left pneumonia with subsegement consolidation.2. Left lung pneumonia with partial consolidation	ADV、HBoV、MP	0	0	1.04E+07	1:1280	0	0
S6	1	1	1.5	Pneumonia, lung consolidation	1. Left lower lung pneumonia with consolidation.2.Bilateral lung pneumonia with multiple segmental atelectasis	ADV、MP	1.7	0	1.98E+04	0	0	0
S7	1	2	10.3	Mycoplasma pneumoniae pneumonia, lung consolidation	1. Left lung pneumonia.2 left lung pneumonia with partial atelectasis	ADV、MP	0	0	6.88E+06	1:640	0	0

Clinical information of 90 cases												
Sample ID	Month	Gender:1 for male, 2 for female	Age(year)	Discharged with Diagnosis	Imaging:1 for the chest X-ray, 2 for CT scan-chest：	Multiple PCR results	DFA（1.RSV 2.FLU A 3.FLU B 4.PIV1 5.PIV2 6.PIV3 7.ADV 8.hMPV)	Real-time RT-PCR for rhinovirus (RV)	Real-time PCR for Mycoplasma pneumonia（MP)	Serum antibodies against M. hyopneumoniae(MP-IgM)	AlveolarLavage Fluid Culture	Underlying diseases
S8	1	2	7.4	Pneumonia, lung consolidation	2. Left lower lung pneumonia with atelectasis,lung consolidation	MP	0	0	8.00E+05	1:160	0	0
S9	1	1	7.1	Influenza (influenza A), severe pneumonia (consolidation), febrile convulsion	1.Bronchopneumonia.2.Bilateral pneumonia, lower left lung atelectasis, bilateral lung pleural effusion	ADV、FLUA	2	0	0	0	0	0
S10	1	1	3.6	Mycoplasma pneumoniae pneumonia, influenza	1.Bilateral pneumonia with partial consolidation.2.Pneumonia, bilateral pleural effusion	ADV、MP、H1N1	2	0	5.95E+07	1:1280	0	0
S11	1	1	1.6	Pneumonia, respiratory syncytial virus infection	1Pneumonia	RSV-A、MP	1.2	0	2.85E+03	1:160	0	0
S12	1	2	7	Mycoplasma pneumoniae pneumonia (associated with rhinovirus infection), lung consolidation	1.Pneumonia, ght lung atelectasis	RV/EV、MP	0	1	/	1:1280	0	0
S13	1	1	3.6	Pneumonia	1.Left lung pneumonia	MP	0	0	/	/	0	0
S14	1	1	12	Pneumonia, lung consolidation	1Pneumonia 2.Pneumonia, lung consolidation	ADV、MP	0	0	0	0	0	0

Clinical information of 90 cases													
Sample ID	Month	Gender:1 for male, 2 for female	Age(year)	Discharged with Diagnosis	Imaging:1 for the chest X-ray, 2 for CT scan-chest:	Multiple PCR results	DFA（1.RSV 2.FLU A 3.FLU B 4.PIV1 5.PIV2 6.PIV3 7.ADV 8.hMPV)	Real-time RT-PCR for rhinovirus (RV)	Real-time PCR for Mycoplasma pneumonia（MP)	Serum antibodies against M. hyopneumoniae(MP-IgM)	AlveolarLavage Fluid Culture	Underlying diseases
S15	1	1	3.8	Mycoplasma pneumoniae pneumonia, lung consolidation	1.Bronchopneumonia.2.Pneumonia and left lower lung consolidation.	MP	0	0	8.23E+07	1:1280	0	0
S16	1	1	3.1	Pneumonia (lung consolidation) (mycoplasma pneumoniae, adenovirus, respiratory syncytial virus), pleural effusion (left)	1. Bilateral peumonia. 2.Left lung pneumonia with consolidation, pleural effusion	MP、HCoV-OC43	2.7	0	9.48E+03	0	0	0
S17	1	1	6	Pneumonia	1.Right lung pneumonia with partial consolidation	ADV、MP	0	0	2.12E+07	1:1280	0	0
S18	1	1	12	Pneumonia,Immunodeficiency disease (PIK3CD mutation), bronchiectasis, fungal infection, proteinuria	1.Bronchopneumonia, bilateral pleural reaction.2.Bilateral bronchiectasis with pneumonia and segmental atelectasis	ADV、MP	0	0	0	/	0	Immune deficiency(PIK3CD gene mutation)
S19	2	1	1.3	Pneumonia, respiratory syncytial virus infection, adenovirus infection, rotavirus enteritis	1.Pneumonia. 2.Pneumonia，left upper lobe subsegmental consolidation	ADV、MP、HCoV-OC43	1.7	0	0	0	0	0
S20	2	1	7	Mycoplasma pneumoniae pneumonia, lung consolidation, pleural effusion	2.The left pneumonia with multiple segement consolidation and atreness, left lung pleural effusion	ADV、FLUA、MP、H1N1	0	0	8.90E+06	1:1280	0	0
S21	2	2	6	Mycoplasma pneumoniae pneumonia, lung consolidation	1.Left lung pneumonia.2.Pneumonia, left upper lobe subsegment atelectasis.	ADV、MP	0	0	1.06E+04	1:1280	0	0

Clinical information of 90 cases													
Sample ID	Month	Gender:1 for male, 2 for female	Age(year)	Discharged with Diagnosis	Imaging:1 for the chest X-ray, 2 for CT scan-chest：	Multiple PCR results	DFA（1.RSV 2.FLU A 3.FLU B 4.PIV1 5.PIV2 6.PIV3 7.ADV 8.hMPV)	Real-time RT-PCR for rhinovirus (RV)	Real-time PCR for Mycoplasma pneumonia（MP)	Serum antibodies against M. hyopneumoniae(MP-IgM)	AlveolarLavage Fluid Culture	Underlying diseases
S22	2	1	0.4	Pulmonary infection, lung consolidation, adenovirus infection, low gamma globulin, infant diarrhea, abnormal liver function, convulsions (cause of convulsions to be determined)	1.Bronchopneumonia.2Pneumonia, right lower lobe partial consolidation and atelectasis, right lung pleural effusion	ADV、MP	7	0	0	0	0	Immune deficiency(agammaglobulinemia)
S23	2	1	4.1	Pneumonia, lung consolidation	1.Right upper lobe pneumonia with partial atelectasis, lung consolidation	ADV、MP	0	0	0	0	0	0
S24	2	1	10	Pneumonia, lung consolidation	1.Right lung pneumonia, lung consolidation	ADV、HBoV、MP	0	0	5.83E+07	1:1280	0	0
S25	2	2	7	Mycoplasma pneumoniae, lung consolidation, acute submandibular adenitis, urticaria, acute gastroenteritis	1.Pneumonia.2.Pneumonias, middle lobe atelectasis, lung consolidation	FLUA、MP	0	0	1.13E+06	1:1280	0	0
S26	2	2	7	Pneumonia, acute gastroenteritis	1.Bronchopneumonia.2.Pneumonia	FLUA、HBoV、MP、H1N1	0	0	0	0	0	0
S27	2	1	6	Mycoplasma pneumoniae pneumonia, lung consolidation	1.Right upper lobe pneumonia with atelectasis.2.Right lung pneumonia with right upper lobe egmental atelectasis	ADV、MP	0	0	2.48E+07	1:640	0	0

Clinical information of 90 cases													
Sample ID	Month	Gender:1 for male, 2 for female	Age(year)	Discharged with Diagnosis	Imaging:1 for the chest X-ray, 2 for CT scan-chest：	Multiple PCR results	DFA（1.RSV 2.FLU A 3.FLU B 4.PIV1 5.PIV2 6.PIV3 7.ADV 8.hMPV)	Real-time RT-PCR for rhinovirus (RV)	Real-time PCR for Mycoplasma pneumonia（MP)	Serum antibodies against M. hyopneumoniae(MP-IgM)	AlveolarLavage Fluid Culture	Underlying diseases
S28	2	1	9	Mycoplasma pneumoniae pneumonia, lung consolidation, pleural effusion (right)	1.Pneumonia with lung consolidation, pleural effusion.2.Bilateral pneumonia, right lower lung consolidation, pleural effusion	FLUA、HBoV、MP、HCoV-NL63/HKU1	0	0	8.08E+06	1:1280	0	0
S29	2	1	9	Mycoplasma pneumoniae pneumonia, lung consolidation	1.Right upper lobe pneumonia with segmentary atelectasis	ADV、MP	0	0	2.35E+06	1:1280	0	0
S30	2	2	2.5	Pneumonia, lung consolidation	1.Right upper lung pneumonia with atelectasis	ADV、MP、H1N1	0	0	0	0	0	0
S31	2	1	2.9	Pneumonia	2. Right upper lobe pneumonia	ADV、MP	0	0	0	1:160	0	0
S32	2	1	9	Mycoplasma pneumoniae pneumonia, lung consolidation, pleural effusion	2.Bilateral pneumonia, right lower lobe consolidation, pleural effusion	FLUA、HBoV、MP	0	0	1.15E+-6	1:1280	0	0
S33	2	2	13	Pneumonia, lung consolidation, (refractory) pneumonia mycoplasma infection, adenovirus infection	1. Right lung pneumonia	ADV、MP	7	0	6.65E+04	1:1280	0	0
S34	2	1	1.3	Pneumonia, lung consolidation, streptococcus pneumoniae infection, pleural effusion, rotavirus enteritis	1. Right lung pneumonia with segmental atelectasis, pleural effusion	ADV、MP	0	0	0	0	Streptococcus pneumoniae	0

Clinical information of 90 cases												
Sample ID	Month	Gender:1 for male, 2 for female	Age(year)	Discharged with Diagnosis	Imaging:1 for the chest X-ray, 2 for CT scan-chest：	Multiple PCR results	DFA（1.RSV 2.FLU A 3.FLU B 4.PIV1 5.PIV2 6.PIV3 7.ADV 8.hMPV)	Real-time RT-PCR for rhinovirus (RV)	Real-time PCR for Mycoplasma pneumonia（MP)	Serum antibodies against M. hyopneumoniae(MP-IgM)	AlveolarLavage Fluid Culture	Underlying diseases
S35	2	1	7	Mycoplasma pneumoniae pneumonia, lung consolidation, pleural effusion	1.Right lung pneumonia with lung consolidation, pleural effusion	ADV、MP	0	0	1.43E+06	1:1280	0	0
S36	2	2	8	Pneumonia, lung consolidation, (refractory) mycoplasma pneumonia infection, adenovirus infection, pleural effusion, hypoproteinemia	1.Pneumonia with consolidation, pleural effusion	ADV、MP	7	0	5.60E+05	1:1280	0	0
S37	2	2	6	Pneumonia, lung consolidation	1.Right upper lobe pneumonia with segmentary consolidation and atelectasis	ADV、MP	0	0	1.37E+05	0	0	0
S38	2	2	8	Pneumonia	1.Pneumonia.2.Bilateral lung pneumonia	ADV、MP	0	0	3.93E+06	1:160	0	0
S39	2	2	0.5	Pneumonia, lung consolidation	1.Right lower lung pneumonia with consolidation.	ADV、PIV3、MP、HCoV-C229E	0	0	/	/	0	0
S40	2	2	6	Immunodeficiency disease (no gamma globulin), pulmonary infection.Chronic diarrhea, abnormal white blood cell (granulocytopenia), hypoproteinemia	1.Pneumonia.2.Pneumonia, with right middle lung atelectasis	ADV、MP	0	0	0	/	0	Immune deficiency(agammaglobulinemia)

Clinical information of 90 cases												
Sample ID	Month	Gender:1 for male, 2 for female	Age(year)	Discharged with Diagnosis	Imaging:1 for the chest X-ray, 2 for CT scan-chest：	Multiple PCR results	DFA（1.RSV 2.FLU A 3.FLU B 4.PIV1 5.PIV2 6.PIV3 7.ADV 8.hMPV)	Real-time RT-PCR for rhinovirus (RV)	Real-time PCR for Mycoplasma pneumonia（MP)	Serum antibodies against M. hyopneumoniae(MP-IgM)	AlveolarLavage Fluid Culture	Underlying diseases
S41	2	2	4	Mycoplasma pneumoniae, lung consolidation, pleural effusion, bronchiectasis, rotavirus enteritis	1.Right lung pneumonia, pleural effusion.2. Pneumonia, right lower lobe multisegmental consolidation and partial bronchodilation	ADV、MP	0	0	>1.00E+08	1:1280	0	0
S42	2	2	2.3	Pneumonia, lung consolidation	1.Bronchopneumonia, lung consolidation.2.Pneumonia, left lung consolidation	ADV、MP	0	0	/	1:1280	/	0
S43	2	1	2.8	Mycoplasma pneumoniae pneumonia, lung consolidation	1.Left lung pneumonia.2.Pneumonia with left upper lung consolidation atelectasis	ADV、MP	0	0	>1.00E+08	1:1280	0	0
S44	2	1	8	Mycoplasma pneumoniae pneumonia, pulmonary consolidation (with pulmonary cavity), pleural effusion, parasitic infection (Metacercaria?)	1.Bronchopneumonia.2..Bilateral pneumonia with consolidation, right lower lobe with cavity, pleural effusion	ADV、MP	0	0	7.38E+03	1:1280	0	0
S45	2	2	6	Pneumonia, lung consolidation	1.Right upper lung pneumonia with segmental lung consolidation	ADV、MP	0	0	6.35E+05	1:1280	0	0
S46	2	2	12	Cystic fibrosis, bronchiectasis, chronic diarrhea (pancreatic exocrine insufficiency), nasal and sinus diseases (sinusitis), influenza (A)	2.Bilateral bronchiectasis with pneumonia	ADV、HBoV、MP	0	0	3.35E+03	0	Pseudomonas aeruginosa	Chronic diarrhea with pancreas exocrine insufficiency

Clinical information of 90 cases												
Sample ID	Month	Gender:1 for male, 2 for female	Age(year)	Discharged with Diagnosis	Imaging:1 for the chest X-ray, 2 for CT scan-chest：	Multiple PCR results	DFA（1.RSV 2.FLU A 3.FLU B 4.PIV1 5.PIV2 6.PIV3 7.ADV 8.hMPV)	Real-time RT-PCR for rhinovirus (RV)	Real-time PCR for Mycoplasma pneumonia（MP)	Serum antibodies against M. hyopneumoniae(MP-IgM)	AlveolarLavage Fluid Culture	Underlying diseases
S47	2	1	2.8	Mycoplasma pneumoniae pneumonia, lung consolidation	1.Left lung pneumonia.2.Pneumonia, left upper lung consolidation and atelectasis	ADV、MP、HCoV-C229E	0	0	>1.00E+08	1:1280	0	0
S48	3	1	0.7	Pneumonia (adenovirus infection), lung consolidation, pleural effusion, liver dysfunction, rotavirus enteritis	1. Inflammation in both lungs.2.Double pneumonitis with partial lung consolidation and pleural effusion	ADV、MP	0	1	0	0	0	0
S49	3	2	8	Mycoplasma pneumoniae pneumonia, lung consolidation (right lower lung), pleural effusion (right), epilepsy	1.Right lung inflammation and pleural effusion	ADV、MP	8	0	3.05E+06	/	0	Epilepsy
S50	3	2	4.6	Pneumonia	1.Bilateral lower lobe pneumonia	ADV、MP	0	0	0	/	0	0
S51	3	1	3.1	Mycoplasma pneumonia, Prader-Willi syndrome	1.Bronchopneumonia	ADV、MP	0	0	0	0	0	Prader - Willi syndrome
S52	3	2	0.7	Mycoplasma pneumoniae pneumonia, lung consolidation	1.Pneumonia.2. Left lower lobe pneumonia, lung consolidation	ADV、MP	0	0	0	1:1280	0	0
S53	3	1	1.7	Immunodeficiency (chronic granulomatous disease), pulmonary infection, diarrhea	1.Bilateral interstitial pneumonia.2.Bilateral interstitial pneumonia	ADV、MP	0	0	0	0	0	Immune deficiency(chronic granulomatous disease)

Clinical information of 90 cases													
Sample ID	Month	Gender:1 for male, 2 for female	Age(year)	Discharged with Diagnosis	Imaging:1 for the chest X-ray, 2 for CT scan-chest：	Multiple PCR results	DFA（1.RSV 2.FLU A 3.FLU B 4.PIV1 5.PIV2 6.PIV3 7.ADV 8.hMPV)	Real-time RT-PCR for rhinovirus (RV)	Real-time PCR for Mycoplasma pneumonia（MP)	Serum antibodies against M. hyopneumoniae(MP-IgM)	AlveolarLavage Fluid Culture	Underlying diseases
S54	3	2	2.5	Tuberous sclerosis, pneumonia, gastroenteritis	1.Pneumonia	FLUA、MP	1	0	0	0	0	Tuberous sclerosis
S55	3	1	2.8	Mycoplasma pneumoniae pneumonia, lung consolidation	1.Left lung pneumonia, with segmentary atelectasis.2.Pneumonia, left upper lobe segmental atelectasis	ADV、MP	0	0	1.84E+04	1:1280	0	0
S56	3	2	6	Pneumonia, lung consolidation (lower left lung)	1.Pneumonia.2.Left lower lobe pneumonia, with atelectasis	ADV、MP	0	0	4.75E+04	/	0	0
S57	3	1	3.7	Severe pneumonia, respiratory failure, rhinovirus infection, lung consolidation, pleural effusion, myocardial damage, liver dysfunction	1.Pneumonia.2.Pneumonia, left lung partial consolidation and atelectasis, bilateral pleural effusion	ADV、MP	0	0	0	0	0	0
S58	3	2	6	Pneumonia, pulmonary consolidation, mycoplasma infection, pleural effusion, pulmonary necrosis, pulmonary embolism, pulmonary cavity	1.Pneumonia, with right lung pleural effusion.2.Right lung pneumonia, middle and lower lobe consolidation,bilateral pleural effusion	ADV、MP	0	0	1.55E+04	1:1280	0	0
S59	3	1	1.3	Pneumonia, lung consolidation	1.Pneumonia, left lower lobe segmental consolidation	MP	0	0	0	0	Streptococcus pneumoniae	0

Clinical information of 90 cases												
Sample ID	Month	Gender:1 for male, 2 for female	Age(year)	Discharged with Diagnosis	Imaging:1 for the chest X-ray, 2 for CT scan-chest：	Multiple PCR results	DFA（1.RSV 2.FLU A 3.FLU B 4.PIV1 5.PIV2 6.PIV3 7.ADV 8.hMPV)	Real-time RT-PCR for rhinovirus (RV)	Real-time PCR for Mycoplasma pneumonia（MP)	Serum antibodies against M. hyopneumoniae(MP-IgM)	AlveolarLavage Fluid Culture	Underlying diseases
S60	3	1	2.5	Pneumonia, lung consolidation	2.Bronchopneumonia, right lower lobe consolidation, right long pleural effusion	MP、HCoV-C229E	0	0	2.46E+05	1:160	0	0
S61	3	1	2.1	Mycoplasma pneumoniae	1.Bronchitis.2.Bilateral lung pneumonia	MP	0	0	7.98E+06	1:640	0	0
S62	3	1	1.3	Pneumococcal pneumonia (with adenovirus), lung consolidation, pleural effusion	1.Right lung pneumonia with pleural effusion on the right.2. Right lowe lobe pneumonia with partial consolidation and atelectasis, leural effusion	ADV、MP、HCoV-C229E	7	0	0	0	0	0
S63	3	1	2.11	Mycoplasma pneumoniae, lung consolidation, rotavirus enteritis	1.Pneumonia.2.Bilateral lung pneumonia with segmental consolidation	ADV、MP	0	0	7.83E+06	1:1280	0	0
S64	3	2	1.9	Pneumonia, lung consolidation, mycoplasma pneumoniae	1.Right lung pneumonia with right upper lobe atelectasis. 2.Pneumonia, right upper lobe consolidation, right middle and lower lobe subsegmental atelectasis	ADV、MP	0	0	3.18E+06	/	0	0
S65	3	1	1.2	Pneumonia (parainfluenza virus infection, adenovirus infection)	1.Pneumonia	ADV、MP、PIV3	6.7	0	0	0	0	0

Clinical information of 90 cases													
Sample ID	Month	Gender:1 for male, 2 for female	Age(year)	Discharged with Diagnosis	Imaging:1 for the chest X-ray, 2 for CT scan-chest：	Multiple PCR results	DFA（1.RSV 2.FLU A 3.FLU B 4.PIV1 5.PIV2 6.PIV3 7.ADV 8.hMPV)	Real-time RT-PCR for rhinovirus (RV)	Real-time PCR for Mycoplasma pneumonia（MP)	Serum antibodies against M. hyopneumoniae(MP-IgM)	AlveolarLavage Fluid Culture	Underlying diseases
S66	3	1	1.1	Bronchopneumonia	1.Bronchopneumonia	ADV、MP、PIV3	0	0	0	0	0	0
S67	3	2	1.2	Pneumonia, lung consolidation, pleural effusion, abnormal liver function, anemia (mild to moderate)	1.Pneumonia, with right lung pleural effusion. 2.Bilateral lung pneumonia with partial subsegmental atelectasis,pleural effusion	ADV、MP	0	0	0	0	0	0
S68	3	1	1.9	Adenovirus pneumonia, lung consolidation	1.Pneumonia. 2.Pneumonia, right lower lobe segmental consolidation	ADV、MP	0	0	0	0	0	0
S69	3	1	6	Influenza (A), Mycoplasma pneumoniae (lung consolidation)	2.Pneumonia, left upper lobe consolidation and atelectatic	ADV	2.7	0	1.83E+05	1:640	0	0
S70	3	2	7	Influenza (A), mycoplasma pneumoniae	1.Pneumonia	ADV、MP	2	0	0	1:1280	0	0
S71	3	1	3.5	Mycoplasma pneumoniae	1.Pneumonia.2.Bilateral lung pneumonia, pleural effusion	ADV、MP	6	1	/	1:1280	0	0

Clinical information of 90 cases												
Sample ID	Month	Gender:1 for male, 2 for female	Age(year)	Discharged with Diagnosis	Imaging:1 for the chest X-ray, 2 for CT scan-chest：	Multiple PCR results	DFA（1.RSV 2.FLU A 3.FLU B 4.PIV1 5.PIV2 6.PIV3 7.ADV 8.hMPV)	Real-time RT-PCR for rhinovirus (RV)	Real-time PCR for Mycoplasma pneumonia（MP)	Serum antibodies against M. hyopneumoniae(MP-IgM)	AlveolarLavage Fluid Culture	Underlying diseases
S72	3	2	12	Mycoplasma pneumoniae pneumonia, lung consolidation, pleural effusion, pericardial effusion	1.Bilateral lung pneumonia.2.Bilateral lung pneumonia, right upper lobe consolidation, pleural effusion, pericardial effusion	PIV3、RV/EV、MP	0	0	6.54E+06	1:1280	0	0
S73	3	1	0.6	Pneumonia, lung consolidation, congenital bronchial stenosis, muscular torticollis, hemangioma	1.Bilateral lung interstitial pneumonia. 2.Bilateral lung pneumonia with bilateral lung multiple subsegmental consolidation, right lung pleural effusion	ADV、hMPV、MP	0	0	0	0	0	Congenital bronchial stenosis muscular torticollis
S74	3	1	4.9	Mycoplasma pneumoniae pneumonia, lung consolidation	1.Right lower lung pneumonia with consolidation.2. Right lower lung pneumonia with consolidation,atelectasis, right lung pleural effusion	ADV、FLUB、MP	0	0	3.03E+06	1:1280	0	0
S75	3	2	7	Mycoplasma pneumoniae pneumonia, lung consolidation, pleural effusion	1.Pneumonia, lung consolidation,bilateral pleural pleural effusion	MP	0	0	3.43E+06	1:1280	0	0
S76	3	2	5.3	Pneumonia, lung consolidation	1.Right puuer lung pneumonia.2.Bilateral lung pneumonia, right upper and left lower lung consolidation	ADV、MP	0	0	2.32E+04	/	0	0

Clinical information of 90 cases												
Sample ID	Month	Gender:1 for male, 2 for female	Age(year)	Discharged with Diagnosis	Imaging:1 for the chest X-ray, 2 for CT scan-chest：	Multiple PCR results	DFA（1.RSV 2.FLU A 3.FLU B 4.PIV1 5.PIV2 6.PIV3 7.ADV 8.hMPV)	Real-time RT-PCR for rhinovirus (RV)	Real-time PCR for Mycoplasma pneumonia（MP)	Serum antibodies against M. hyopneumoniae(MP-IgM)	AlveolarLavage Fluid Culture	Underlying diseases
S77	3	1	6	Bronchial asthma (non-critical), mycoplasma pneumoniae, lung consolidation	1. Left lung pneumonia, left lower lobe segmentary consolidation, left lung pleural effusion	ADV、MP	0	0	8.13E+04	1:320	0	Bronchial asthma
S78	3	1	9	Influenza (A), pneumonia (lung consolidation)	1.Right middle lobe pneumonia and consolidation atelectasis	ADV、MP、PIV2	2	0	1.72E+07	0	0	0
S79	3	2	8	Influenza a, pneumonia (Staphylococcus aureus)	1.Right upper and lower lung pneumonia.2.Pneumonia, right upper lobe and left lower lobe segmental consolidation atelectasis	ADV、MP	0	0	0	0	Staphylococcus aureus	0
S80	3	1	2.4	Pneumonia, lung consolidation, mycoplasma pneumoniae infection, adenovirus infection	1.Pneumonia, consolidation,segmentary atelectasis	ADV、MP、HCoV-NL63/HKU1	7	0	0	1:1280	0	0
S81	3	1	0.8	Pneumonia, lung consolidation, adenovirus infection	1.Pneumonia.2.Pneumonia, partial consolidation	ADV、MP、HCoV-NL63/HKU1	7	0	0	0	0	0
S82	3	1	6	Pneumonia, lung consolidation	1.Pneumonia, left lower lobe partial consolidation	ADV、FLUA、MP	0	0	8.80E+05	/	0	0

Clinical information of 90 cases												
Sample ID	Month	Gender:1 for male, 2 for female	Age(year)	Discharged with Diagnosis	Imaging:1 for the chest X-ray, 2 for CT scan-chest：	Multiple PCR results	DFA（1.RSV 2.FLU A 3.FLU B 4.PIV1 5.PIV2 6.PIV3 7.ADV 8.hMPV)	Real-time RT-PCR for rhinovirus (RV)	Real-time PCR for Mycoplasma pneumonia（MP)	Serum antibodies against M. hyopneumoniae(MP-IgM)	AlveolarLavage Fluid Culture	Underlying diseases
S83	3	2	3	Pneumonia (metapneumovirus), lung consolidation, (right) unilateral emphysema, mycoplasma pneumoniae infection, vulva and vaginal candidiasis	1.Left lung pneumonia,subsegmentary atelectasis.2.Bilateral lung pneumonia, right upper and lower lobe segmental consolidation, atelectasis, right lung and left lower	ADV、HCoV-NL63/HKU1	0	0	0	1:1280	0	0
S84	3	1	4.8	Mycoplasma pneumoniae pneumonia, lung consolidation	1.Pneumonia, right upper lung atelectasis	ADV、MP、HCoV-C229E	0	0	1.63E+06	1:1280	0	0
S85	3	1	3.1	Pneumonia (adenovirus), lung consolidation, pleural effusion (left), vascular malformation	1.Bronchopneumonia.2.Right lung pneumonia, left lower lobe consolidation, left lunf pleural effusion	ADV、MP	0	0	2.40E+04	0	0	0
S86	3	2	5.1	Mycoplasma pneumoniae pneumonia, lung consolidation, pleural effusion, liver dysfunction, hypoproteinemia	1.Left lower lung pneumoniae pneumonia, lung consolidation, pleural effusion	ADV、MP、PIV2	0	0	2.27E+07	1:1280	0	0
S87	3	1	3.7	Pneumonia (rhinovirus, mycoplasma infection), congenital malformation (absence of single kidney)	1.Pneumonia	MP、HCoV-C229E	0	1	4.13E+03	1:1280	0	Congenital malformation (single kidney deficiency)
S88	3	2	7	Pneumonia, atelectasis	1.Bronchitis.2.Pneumonia, left upper lobe subsegmental atelectasis	ADV、MP、PIV2	0	0	2.31E+04	/	0	0

Clinical information of 90 cases													
Sample ID	Month	Gender:1 for male, 2 for female	Age(year)	Discharged with Diagnosis	Imaging:1 for the chest X-ray, 2 for CT scan-chest：	Multiple PCR results	DFA（1.RSV 2.FLU A 3.FLU B 4.PIV1 5.PIV2 6.PIV3 7.ADV 8.hMPV)	Real-time RT-PCR for rhinovirus (RV)	Real-time PCR for Mycoplasma pneumonia（MP)	Serum antibodies against M. hyopneumoniae(MP-IgM)	AlveolarLavage Fluid Culture	Underlying diseases
S89	3	1	6	Mycoplasma pneumoniae pneumonia, lung consolidation, pleural effusion, cough variant asthma	2.Bilateral lung pneumonia, right lower lobe consolidation and atelectasis, bilateral lung pleural effusion	ADV、MP	0	0	1.34E+04	1:160	0	0
S90	3	1	0.8	Pneumonia, lung consolidation, liver dysfunction, congenital heart disease (after surgery)	1.Pneumonia, consolidation, bilateral lung effusion.2.Bilateral lung pneumonia, multiple segmental lung consolidation; bilateral lung pleural reaction	ADV、MP、HCoV-OC43	7	0	0	0	0	Congenital heart disease (postoperative)
S91	3	2	7	Mycoplasma pneumoniae, viral infection (metapneumovirus), lung consolidation, liver dysfunction	1.Right lower lung pneumonia, consolidation	ADV、MP	8	0	4.13E+06	1:1280	0	0

1.S43 and S47 were from one same case;2."/" for no examination;3.MP-IgM was detected by an ELISA method(Human Mycoplasma pneumoniae antibody ELISA kit, Wuhan Yipu Biotechnology Co.Ltd, Wuhan, China).

Table S1

Results of 91 samples tested by RespiFinder 2SMART Multi-PCR and Conventional Tests

pathogen	No. of Multi-PCR / CT	pathogens	No. of Multi-PCR / CT	pathogens	No. of Multi-PCR / CT	pathogens	No. of Multi-PCR / CT
**MP**	6/42	MP, ADV	47/2	MP, ADV, FLUA	1/2	MP, ADV, PIV3, HCoV-C229E	1/0
**ADV**	1/5	MP, RV	1/2	MP, RV, PIV3	1/0	MP, ADV, FLUA, H1N1-2009	1/0
**RV/EV**	0/2	MP, FLUA	2/2	ADV, FLUA, hMPV	0/0	MP, FLUA, HBoV, HCoV-NL63/HKU1	1/0
**RSV**	0/1	MP, hMPV	1/2	MP, FLUA, RSV	0/1	MP, FLUA, HBoV, H1N1-2009	1/0
**FLUA**	0/2	MP, PA	0/1	MP, ADV, RSV	0/1		
**SP**	0/2	ADV, RSV	1/1	MP+ADV+PIV2	3/0		
**SA**	0/1	ADV, PIV3	0/1	MP, ADV, HCoV-C229E	3/0		
		MP, HCoV-OC43	2/0	MP, ADV, HCoV-OC43	2/0		
		MP, HCoV-C229E	2/0	MP, ADV, HCoV-NL63/HKU1	2/0		
		ADV, FLUA	1/0	MP, ADV, H1N1-2009	2/0		
		ADV, HCoV-NL63/HKU1	1/0	MP, ADV, HBoV	3/0		
		RV, PIV3	0/1	MP, ADV, PIV3	2/0		
				MP, ADV, FLUB	1/0		
				MP, ADV, hMPV	1/0		
				MP, FLUA, HBoV	1/0		
Unique pathogen (n=7/55)		Dual pathogens (n=58/12)		Triple pathogens (n=22/4)		Quadruple pathogens(n=4/0)	

(PA：Pseudomonas aeruginosa；SA, Staphylococcus aureus; SP, Streptococcus pneumonia; The Multi-PCR targeted pan-RV/EV).

Table S2

Sensitivity and specificity of RespiFinder 2SMART Multi-PCR compared with DFA for 8 viruses.

	DFA for 8 viruses		
	Positive(n)	Negative(n)	Total(n)
**Multi-PCR**			
**Positive (n)**	20	60	80
**Negative (n)**	1	10	11
**Total (n)**	21	70	91
**Positive Predictive Value (%)**	25.00（95%CI，16.28-36.15）		
**Negative Predictive Value (%)**	90.91（95%CI，57.12-99.52）		
**Sensitivity (%)**	95.23（95%CI，74.13-99.75）		
**Specificity (%)**	14.39（95%CI，7.43-25.17）		

Table S3

Sensitivity and specificity of RespiFinder 2SMART Multi-PCR compared with Real-time PCR for MP and DFA for ADV.

	Real-time PCR for MP			DFA for ADV		
	Positive(n)	Negative(n)	Total(n)	Positive(n)	Negative(n)	Total(n)
**Multi-PCR**						
**Positive (n)**	54	29	83	11	61	72
**Negative (n)**	1	2	3	1	18	19
**Total (n)**	55	31	86	12	79	91
**Positive Predictive Value (%)**	65.06（95%CI，53.72-74.98）			15.27（95CI，8.23-26.11）		
**Negative Predictive Value (%)**	66.67（95%CI，12.53-98.23）			94.73（95%CI，71.89-99.72）		
**Sensitivity (%)**	98.18（95%CI，89.00-99.90）			91.66（95%CI，59.75-99.56）		
**Specificity (%)**	6.45（95%CI，1.12-22.84）			22.78（95%CI，14.40-33.85）		

Table S4

Sensitivity and specificity of the RespiFinder 2SMART Multi-PCR compared with CT for pathogens (8 viruses, RV/EV and MP).

	CT		
	Positive (n)	Negative (n)	Total (n)
**Multi-PCR**			
**Positive (n)**	69	22	91
**Negative (n)**	0	0	0
**Total (n)**	69	22	91
**Positive Predictive Value (%)**	73.17（95%CI，62.05-82.08）		
**Negative Predictive Value (%)**	-		
**Sensitivity (%)**	100（95%CI，81.50-100）		
**Specificity (%)**	0.00（95%CI，0.00-6.57）		
